# The ReHand-BCI trial: a randomized controlled trial of a brain-computer interface for upper extremity stroke neurorehabilitation

**DOI:** 10.3389/fnins.2025.1579988

**Published:** 2025-06-18

**Authors:** Jessica Cantillo-Negrete, Martín Emiliano Rodríguez-García, Paul Carrillo-Mora, Oscar Arias-Carrión, Emmanuel Ortega-Robles, Marlene A. Galicia-Alvarado, Raquel Valdés-Cristerna, Ana G. Ramirez-Nava, Claudia Hernandez-Arenas, Jimena Quinzaños-Fresnedo, Ma. del Refugio Pacheco-Gallegos, Norma Marín-Arriaga, Ruben I. Carino-Escobar

**Affiliations:** ^1^Technological Research Subdirection, Instituto Nacional de Rehabilitacion Luis Guillermo Ibarra Ibarra, Mexico City, Mexico; ^2^Electrical Engineering Department, Universidad Autonoma Metropolitana - Iztapalapa, Mexico City, Mexico; ^3^Division of Research in Clinical Neuroscience, Instituto Nacional de Rehabilitacion Luis Guillermo Ibarra Ibarra, Mexico City, Mexico; ^4^Unidad de Trastornos de Movimiento y Sueño (TMS), Hospital General Dr Manuel Gea Gonzalez, Mexico City, Mexico; ^5^Experimental Neurology, Instituto Nacional de Rehabilitacion Luis Guillermo Ibarra Ibarra, Mexico City, Mexico; ^6^Division of Neurological Rehabilitation, Instituto Nacional de Rehabilitacion Luis Guillermo Ibarra Ibarra, Mexico City, Mexico; ^7^Division of Imaging, Instituto Nacional de Rehabilitacion Luis Guillermo Ibarra Ibarra, Mexico City, Mexico

**Keywords:** brain-computer interface, stroke, EEG, robot, magnetic resonance imaging, transcranial magnetic stimulation

## Abstract

**Background:**

Brain-computer interfaces (BCI) are a promising complementary therapy for stroke rehabilitation due to the close-loop feedback that can be provided with these systems, but more evidence is needed regarding their clinical and neuroplasticity effects.

**Methods:**

A randomized controlled trial was performed using the ReHand-BCI system that provides feedback with a robotic hand orthosis. The experimental group (EG) used the ReHand-BCI, while sham-BCI was given to the control group (CG). Both groups performed 30 therapy sessions, with primary outcomes being the Fugl-Meyer Assessment for the Upper Extremity (FMA-UE) and the Action Research Arm Test (ARAT). Secondary outcomes were hemispheric dominance, measured with electroencephalography and functional magnetic resonance imaging, white matter integrity via diffusion tensor imaging, and corticospinal tract integrity and excitability, measured with transcranial magnetic stimulation.

**Results:**

At post-treatment, patients in both groups had significantly different FMA-UE scores (EG: baseline = 24.5[20, 36], post-treatment 28[23, 43], CG: baseline = 26[16, 37.5], post-treatment = 34[17.3, 46.5]), while only the EG had significantly different ARAT scores at post-treatment (EG: baseline = 8.5[5, 26], post-treatment = 20[7, 36], CG: baseline = 3[1.8, 30.5], post-treatment = 15[2.5, 40.8]). In addition, across the intervention, the EG showed trends of more pronounced ipsilesional cortical activity and higher ipsilesional corticospinal tract integrity, although these differences were not statistically different compared to the control group, likely due to the study’s sample size.

**Conclusion:**

To the authors’ knowledge, this is the first clinical trial that has assessed such a wide range of physiological effects across a long BCI intervention, implying that a more pronounced ipsilesional hemispheric dominance is associated with upper extremity motor recovery. Therefore, the study brings light into the neuroplasticity effects of a closed-loop BCI-based neurorehabilitation intervention in stroke.

**Clinical trial registration:**

https://clinicaltrials.gov/, identifier NCT04724824.

## 1 Introduction

Stroke is among the leading causes of disability. It has an estimated global prevalence of 89.13 million cases and a global incidence of 11.71 million cases per year (Tsao et al., 2023). Hemiparesis, one of stroke’s sequelae, is comprised by the complete or partial paralysis of one hemibody and is one of the most important contributors of stroke patients’ disability. Furthermore, approximately 60% of patients with severe upper extremity paralysis will be unable to perform activities of daily living with their paralyzed limb after 6 months of conventional rehabilitation (Hatem et al., 2016). For these reasons, experimental interventions for upper extremity rehabilitation in stroke have been proposed, such as functional electrical stimulation (FES) (Kristensen et al., 2022), robot-assisted training (Yang et al., 2023), repetitive transcranial magnetic stimulation (Zhang et al., 2017), and brain-computer interfaces (BCI) (Mansour et al., 2022). Particularly, BCI are systems that can allow stroke patients to control external devices, by decoding patients’ motor intention (MI) of their paralyzed upper extremity using information extracted from the central nervous system. Due to being non-invasive, most of the BCI that have been applied for upper extremity rehabilitation in stroke use electroencephalography (EEG) for acquiring information of the brain cortex (Cervera et al., 2018; Zhang et al., 2024). It has been hypothesized that the closed-loop feedback provided by a BCI promotes neuroplasticity, the primary recovery mechanism in stroke (Belkacem et al., 2023).

Clinical trials comprising BCI interventions that incorporate robotic rehabilitation devices as feedback have shown promising clinical results (Cervera et al., 2018; López-Larraz et al., 2018; Mansour et al., 2022). However, the mechanisms of upper extremity stroke recovery during BCI interventions remain largely unknown, with advanced imaging techniques, such as functional magnetic resonance imaging (fMRI), providing the best opportunity to understand these mechanisms (Qu et al., 2024). Moreover, it has been reported that more clinical trials are still needed to assess the efficacy of BCI interventions for upper extremity stroke rehabilitation (Cervera et al., 2018; López-Larraz et al., 2018; Qu et al., 2024; Zhang et al., 2024). Particularly, differences in clinical recovery and neuroplastic changes between stroke patients that had upper extremity BCI interventions with robotic rehabilitation devices as feedback, compared to the effects of control groups that received therapy only with the robotic device, have not yet been sufficiently reported (Qu et al., 2024). To the authors’ knowledge, only Ramos-Murguialday et al. (2013) have evaluated the effects of a BCI with a hand robotic device as feedback in stroke patients with severe upper extremity disability, using, amongst other clinical and physiological variables, fMRI, and compared the experimental groups’ recovery to a control group that received a sham BCI intervention. Therefore, more evidence from clinical trials is needed to evaluate if upper extremity motor recovery is enhanced with BCI rehabilitation due to the effects of closed-loop feedback, compared to only applying robotic rehabilitation devices without BCI control.

This study presents the results of the ReHand-BCI clinical trial, aimed at assessing the efficacy and neuroplastic effects of a BCI intervention for upper extremity stroke rehabilitation by comparing the recovery observed in an experimental group, that received therapy using a BCI-controlled robotic device, with a control group, that received a sham-BCI therapy. For assessing neuroplasticity, advanced imaging and electrophysiological techniques were used. To the authors’ knowledge, this is the first reported BCI-based clinical trial that has compared neuroplasticity effects between an experimental and a sham intervention using fMRI, diffusion tensor imaging (DTI), EEG, and transcranial magnetic stimulation (TMS). The hypothesis of the study was that the experimental group would have a higher recovery of upper extremity function, higher ipsilesional cortical activity, higher ipsilesional corticospinal tract integrity and corticospinal tract excitability, compared to the control group.

## 2 Materials and methods

### 2.1 Study design

This study was approved by the Research Committee and the Ethics Committee of the National Institute of Rehabilitation Luis Guillermo Ibarra Ibarra (25/19 AC) and was conducted in accordance with the Declaration of Helsinki. Written informed consent, approved by the Ethics Committee of the National Institute of Rehabilitation Luis Guillermo Ibarra Ibarra, was obtained from all participants. The study was prospectively registered on January 26th of 2021 in ClinicalTrials.gov (Registration number: NCT04724824, Name: Validation of a Brain-Computer Interface for Stroke Neurological Upper Limb Rehabilitation). The study was a single center, triple-blinded randomized controlled clinical trial performed at the National Institute of Rehabilitation Luis Guillermo Ibarra Ibarra. The study blinding was performed by concealing patients’ group allocation to the personnel that performed the BCI therapies, to the clinicians that performed the outcome assessments and statistical analyzes, and to the patients that participated in the study. The planned sample was of 15 patients in the experimental and 15 patients in the control group. Sample size was chosen to achieve a statistical power higher than 80% for intergroup analysis of MRI-derived functional and structural features (Desmond and Glover, 2002; Heiervang et al., 2006). A block randomization was performed in Microsoft Excel. Participants were randomly allocated to one of two interventions comprising the experimental or the control group, with a 1:1 allocation ratio. In the experimental group, participants underwent 30 sessions of BCI therapy using the ReHand-BCI system, with patients controlling a robotic hand orthosis with their affected hand’s MI. In the control group, participants underwent 30 sessions of a sham-BCI therapy, using the ReHand-BCI system in “Sham mode,” where participants attempted to control the robotic hand orthosis with their affected hand’s MI, but the orthosis was randomly activated. Therefore, feedback was independent of the participant’s movement intention.

### 2.2 Participants

Patients who met the following criteria participated in the study. Inclusion criteria: adults (>18 years); any sex; diagnosed by a neurologist with a first ischemic or hemorrhagic stroke in either hemisphere with diagnosis confirmation through neuroimaging studies; time since stroke onset within the range of 3 to 24 months; diagnosed hand paresis (Motricity index from 0 to 22 Bohannon, 1999); right-handed before the stroke, to prevent cortical activity variability due to hand dominance; normal or corrected to normal vision; had at most mild alterations in attention and memory processes according to the neuropsychological test NEUROPSI (Ostrosky et al., 2019); and with no previous diagnosed neurological diseases. Exclusion criteria: clinical diagnosis of severe hand spasticity (Modified Ashworth Scale (Chen et al., 2020) > 2); severe aphasia; severe depression; severe attention deficits; previous diagnosis of traumatic brain injury, spinal cord injury or peripheral nerve injury; having a pacemaker or metal implants incompatible with magnetic resonance; or prescriptions of medicaments that interfere with recovery or cognitive performance. Elimination criteria: patients’ determination to withdraw from the study; patients not attending to at least 80% of the study’s therapy sessions; missing an appointment of an outcome evaluation; epilepsy, seizures, excessive blinking, or movement artifacts during EEG recordings; or symptoms of any other neurological disorder during participation in the study.

### 2.3 ReHand-BCI system

The ReHand-BCI consists of an acquisition, a processing, a feedback stage, and a computer monitor to provide instructions to the patient. In the acquisition stage, 16 active g. LadyBird electrodes (g.tec medical engineering GmbH, Austria), located in the positions F3, FC3, C5, C3, C1, CP3, P3, FCz, Cz, F4, FC4, C6, C4, C2, CP4, and P4 of the 10–10 system, were fixed to a cap to record EEG. The reference electrode was placed in the right earlobe and the ground electrode in the AFz position. A g.USBamp amplifier (g.tec medical engineering GmbH, Austria) acquired EEG at 256 Hz with a 24-bit A/D resolution. The processing stage operated in two modes, a calibration (offline) and a therapy (online) mode. In the calibration mode, pre-recorded EEG data from the previous therapy session was used, except for the first therapy session, for which an EEG baseline recording was used. Pre-recorded EEG data was band-pass filtered with a filter bank (8–12, 12–16, 16–20, 20–24, 24–28, and 28–32 Hz) and notch filtered at 60 Hz with 30th order FIR filters. Logarithmic variance features were then extracted with the Common Spatial Patterns (Blankertz et al., 2008) (CSP) algorithm using 1-s windows from each channel, filtered frequency band, and trial. This procedure encompassed the Filter Bank Common Spatial Patterns algorithm (Ang et al., 2012). Afterwards, relevant extracted CSP features were selected using Particle Swarm Optimization (PSO) and used to train a Linear Discriminant Analysis (Shi and Eberhart, 1998) (LDA) classifier. With the selected subject-specific frequency bands, computed spatial filters, and LDA coefficients obtained in the calibration mode, the therapy mode could classify 1-s windows acquired online as either hand MI or as rest with eyes open period (rest). The processing stage was implemented in a Precision 5820 workstation (Dell Inc., Texas, USA) through a Graphical User Interface programmed in MATLAB (MathWorks Inc., Massachusetts, USA). The feedback stage was comprised by a robotic hand orthosis, connected via Bluetooth to the processing stage, that provided passive finger flexion or extension to the patients’ paralyzed hand. The orthosis was activated by patients’ hand MI in the experimental group and was randomly activated for patients in the control group. A preliminary version of the ReHand-BCI was assessed in a pilot study with a stroke sample (Cantillo-Negrete et al., 2021) and the feedback stage of the system was tested in a healthy population before being used in the present study (Carino-Escobar et al., 2023). A detailed technical description of the ReHand-BCI can be found in previous studies (Cantillo-Negrete et al., 2018; Carino-Escobar et al., 2023). The ReHand-BCI and its operation are depicted in Supplementary Video S1.

### 2.4 BCI therapy and sham-BCI therapy

**Figure 1:**
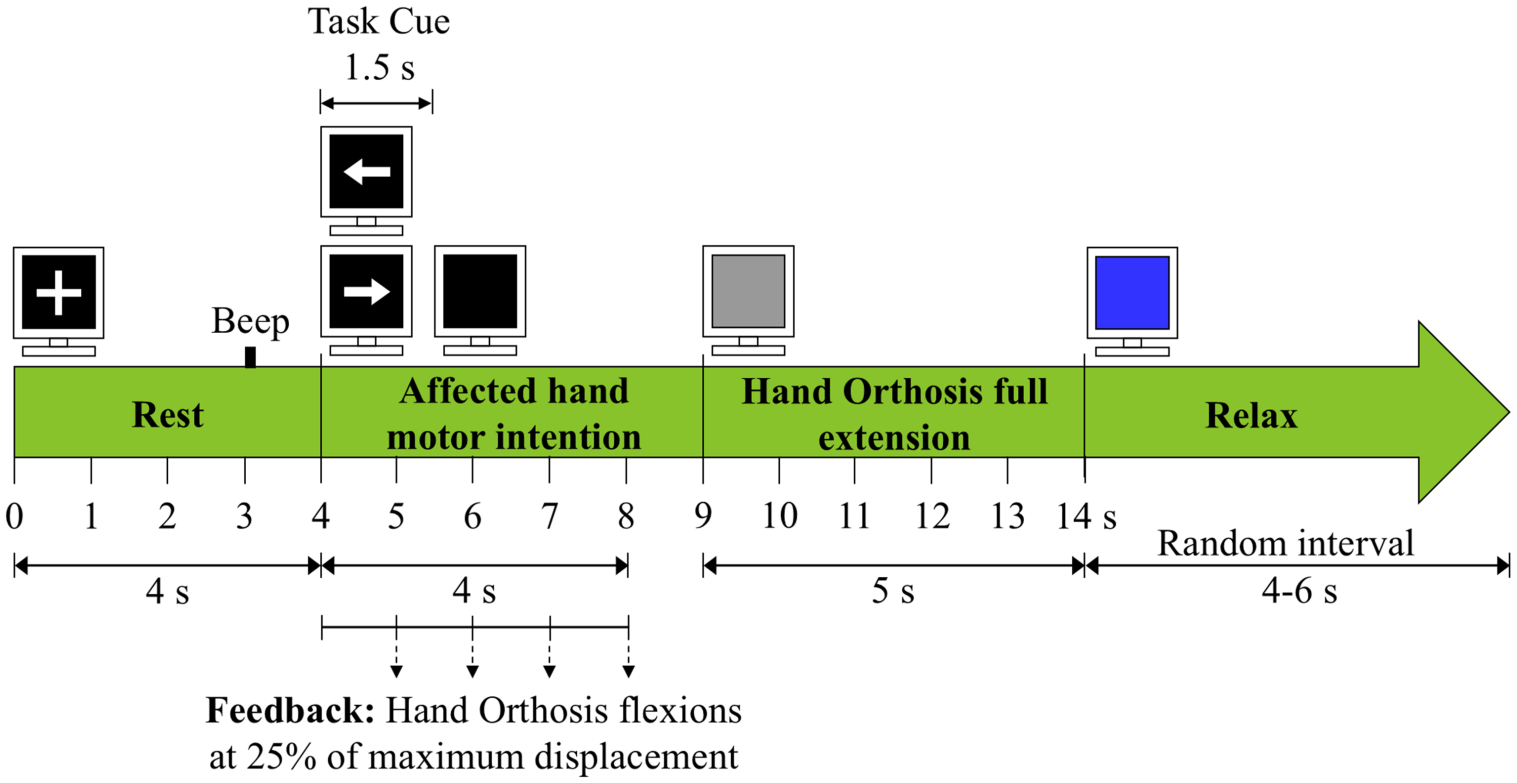
Trial structure for the BCI and the sham-BCI interventions.

Each patient underwent 30 planned sessions of BCI or sham-BCI therapy depending on the assigned group. Five sessions were given per week for 6 weeks. In each therapy session, patients performed 80 trials divided into 4 runs of 20 trials each. Patients were allowed to rest for at least 1 min between runs. BCI and sham-BCI therapy sessions were conducted in the same sound-attenuated room and by the same team of researchers for each patient. Patients sat in a comfortable armchair during therapies. The ReHand-BCI system was set with the robotic orthosis fixed to the patients’ paralyzed hand, and the EEG cap placed over the patients’ head. The computer monitor of the ReHand-BCI was placed in front of the patients at approximately 1.5 m, and a baseball was located under the patients’ paralyzed hand, fixed to one of the arms of the chair. In each session, patients were instructed to perform a series of tasks, constituting a trial, in accordance with visual and auditory cues shown in the monitor. First, a white cross appeared in the monitor that lasted 4 s; during this time, patients were instructed to observe the white cross without performing any other physical or mental activity; encompassing the rest period. Three seconds after the trials’ onset, a beep sound was played by the monitor’s speakers, alerting the patient of the upcoming MI task. Four seconds after the trial’s onset, a white arrow pointing towards the patients’ paralyzed hand appeared in the monitor, indicating to commence the MI of their paralyzed hand. The arrow lasted 1.5 s on the monitor and afterwards disappeared leaving a black screen for another 3.5 s. Patients were instructed to attempt to hold the baseball below their paralyzed hand during the 1.5 s of the arrow appearance and the 3.5 s of the black screen, conforming the MI period. In the experimental group, during the MI period, the orthosis provided passive finger flexion movement at 25% of its maximum displacement (approximately 1.38 cm), whenever the system classified as MI one of the four 1-s windows that comprised the first 4 s of the MI period. Therefore, a maximum of four flexion movements could be elicited per trial. In the control group, the orthosis movement was independent of the system’s recognition of hand MI and was randomly provided according to a random distribution that ranged between a 55 to 85% probability of the orthosis activating in the MI period, with this probability changing in each run. This probability was computed from the range of observed BCI performances achieved by a stroke sample in a preliminary study with an earlier version of the ReHand-BCI (Cantillo-Negrete et al., 2021). After the MI period, the monitor turned grey and, if the participant received finger flexion during the MI period, the orthosis performed finger extension movement, with patients instructed to attempt to open and relax their paralyzed hands, and this period lasted from the 9th to the 14th second of the trial. Finally, the monitor turned blue after 14 s from the trial’s onset, with patients allowed to move, blink, and relax. The blue screen on the monitor lasted randomly between 4 and 6 s to prevent habituation. At the end of each run, a face with different colors and smiling expressions was shown to the patient, indicating the degree of success in controlling the orthosis during the run. Figure 1 shows the time structure of the trials acquired in the therapies. A depiction of the therapy provided with the ReHand-BCI can be found in Supplementary Video S1.

### 2.5 Outcome measures

The primary outcome measures were changes of upper extremity sensorimotor function assessed with the Fugl-Meyer Assessment for the Upper Extremity (FMA-UE) (See et al., 2013) and the assessment of upper extremity functional performance with the Action Research Arm Test (ARAT) (Lang et al., 2006). FMA-UE and ARAT have been used as primary outcomes in studies that assessed BCI efficacy for stroke rehabilitation (Ramos-Murguialday et al., 2013; Ang et al., 2014; Frolov et al., 2017; Wang et al., 2024). Secondary outcomes were the laterality coefficient (LC) derived from EEG, the laterality index (LI) derived from fMRI, the ratio of fractional anisotropy (rFA) derived from DTI, and changes in corticospinal tract (CST) integrity and excitability elicited by TMS. All outcomes were assessed at the onset of the intervention (T0), after the first 15 sessions of the intervention (T1), at the end of the intervention (T2), and 6 months after the baseline measurement (T3). Assessments were performed over 4 to 5 days, and on those days therapy sessions were not provided to patients. The order of the studies was different depending on patients and medical devices availability. The same expert clinician performed all outcome assessments for each patient. Between T0 and T2, patients did not receive conventional therapy focused on their upper extremity, while lower extremity and language therapies were not restricted. From T2 to T3, there were no restrictions on the administration of conventional therapy.

#### 2.5.1 Laterality coefficient

The LC was used to estimate the hemispheric dominance (Kaiser et al., 2012). To compute the LC, EEG recordings were acquired using two interconnected g.USBamp amplifiers and 32 g.SCARABEO active electrodes. Sixteen electrodes were located at the same positions as those used in a therapy session. The other 16 electrodes were located at the F1, F2, FC5, FC6, FC1, FC2, CP5, CP6, CP1, CP2, P1, P2, Fpz, Fz, CPz, and Pz positions of the 10–10 system. The ground and reference electrodes were located at the AFz position and in the right earlobe, respectively. EEG recordings were comprised by 8 runs of 20 trials each, with 4 runs of motor execution of the unaffected hand that were interleaved with 4 runs of MI of the affected hand. Patients rested for 2 min between runs. For this assessment, patients did not wear the robotic orthosis. During the MI period of each trial, patients were instructed to perform (or attempt to perform) continuous finger flexion and extension of the corresponding hand. The trials’ time structure was the same as that of the therapy sessions.

For EEG preprocessing, 10th order FIR temporal filters were applied, including a 7 Hz high-pass filter, a 35 Hz low-pass filter, and a 60 Hz notch filter. Afterwards, an independent component analysis (ICA) and a common average reference spatial filter were used to remove blinking and muscle artifacts, and to reduce the reference location’s effects, respectively (Bertrand et al., 1985). Lastly, a visual inspection was performed to manually remove trials with excessive artifacts.

After preprocessing, time-varying power was obtained after using complex Morlet wavelets on the EEG preprocessed signals. The wavelet transform used a family ratio of 6 and was applied on the 0 to 9 s (0.1 s resolution) window of the trials’ EEG data from 8 to 30 Hz (0.5 Hz resolution). This analysis was performed using the FieldTrip toolbox (Oostenveld et al., 2011) (Radboud University, Nijmegen, the Netherlands, v20181231). Next, event-related desynchronization/synchronization (ERD/ERS) (Pfurtscheller and Lopes da Silva, 1999) was computed and a bootstrapping process was performed to obtain the statistically significant ERD/ERS (sERD/sERS) values in the 4.5 to 8.5 s window of the trials’ time structure, using 1,000 bootstrap samples and a 0.05 critical value (Graimann et al., 2002). The rest period’s 0.5 to 4 s window was used as the normalization reference.

The sERD values were summed at the EEG channels surrounding the motor cortex, encompassing the FC5-FC6, FC3-FC4, FC1-FC2, C5-C6, C3-C4, C1-C2, CP5-CP6, CP3-CP4, and CP1-CP2 channel pairs, to compute the ipsilesional (sERD_IH_) and the contralesional (sERD_CH_) hemispheric desynchronization. The electrodes located on the left or on the right hemisphere were used in the computation of either the ipsilesional or the contralesional desynchronization value depending on the lesion location of each patient. The LC was then obtained as follows:


$$\mathrm{LC}=\frac{\mathrm{sER}D_{\mathrm{IH}}-\mathrm{sER}D_{\mathrm{CH}}}{\mathrm{sER}D_{\mathrm{IH}}+\mathrm{sER}D_{\mathrm{CH}}}$$

An LC was computed separately in the alpha and beta frequency bands given the existing association between motor tasks and desynchronization in these EEG rhythms (Pfurtscheller and Lopes da Silva, 1999; Ang et al., 2015b) and for motor execution of the unaffected hand and MI of the affected hand. LC values of −1 or 1 indicated a complete hemispheric dominance of the contralesional or the ipsilesional hemisphere during the motor task, respectively. An LC value of 0 denoted a symmetrical cortical activity pattern.

#### 2.5.2 Laterality index

The LI was also used to measure the hemispheric dominance during the movement of the patients’ unaffected hand and MI of the affected hand. MRI studies were acquired in a 3 T Philips Ingenia scanner (Philips Medical System, Best, Netherlands). A T1-weighted (T1w) scan, two fMRI sequences, and a DTI sequence (to calculate the rFA, explained below) were obtained at each study. Before the MRI studies, patients were given indications and instructed to remain still. Small cushions were positioned around the patients’ heads to minimize head movement.

The T1w image was obtained with a gradient echo sequence with the following parameters: voxel dimensions = 1 × 1.5 × 1 mm, TR = 6.8 ms, TE = 3.5 ms, flip angle = 8°, FOV = 240 mm, matrix size = 240 × 240, number of slices = 220. The fMRI sequences were acquired via a single-shot echo-planar imaging (EPI) technique with the following parameters: voxel dimensions = 2.38 × 2.4 × 4 mm, TR = 3,000 ms, TE = 35 ms, flip angle = 90°, FOV = 230 mm, matrix size = 96 × 96, number of slices = 36, interslice gap = 0 mm. These functional sequences followed a block design paradigm, comprised by 6 rest blocks alternating with 6 task blocks, where each block lasted 30 s. Patients were instructed to perform continuous finger flexion and extension during each task block, using their unaffected hand during the first fMRI sequence and attempted to perform the same movement with their affected hand during the second sequence. Visual cues were provided during the first 1.5 s of each block by synchronizing the scanner’s pulses via the MiniUSB Trigger interface (Current Designs, Pennsylvania, USA) with a program developed in the PsychoPy software (Peirce et al., 2019) (University of Nottingham, Nottingham, United Kingdom, v2021.2.5). A white cross indicated a rest block while a white arrow pointing to either the patients’ unaffected or affected hand signaled a task block. Finally, the DTI sequence was acquired via a single-shot spin-echo EPI technique with the following acquisition parameters: voxel dimensions = 2.5 × 2.5 × 2.5 mm, TR = 4,035 ms, TE = 83 ms, flip angle = 90°, FOV = 224 mm, matrix size = 90 × 90, number of slices = 72. Here, 15 diffusion-weighted directions (b-value = 800 s/mm^2^) were acquired alongside a non-diffusion-weighted image (b-value = 0 s/mm^2^).

fMRI preprocessing and intrasubject analysis was performed with the FSL software (The University of Oxford, Oxford, United Kingdom, v6.0.4 Jenkinson et al., 2012). Image preprocessing was comprised by realignment, outlier detection, smoothing (full width at half maximum of 5 mm), high-pass filtering (cutoff frequency of 0.011 Hz), and a probabilistic ICA spatial filter (Beckmann and Smith, 2004; Rodríguez-García et al., 2024). Next, a registration to standard MNI152 space was performed by combining a linear registration of the functional images to the native T1w space (Greve and Fischl, 2009) and a non-linear registration from the native T1w space to the MNI152 T1w space (2 mm resolution). Finally, the inverse transformation (invRegF) was computed as the inverse matrix of the combined transformation.

Intrasubject analysis was performed via a generalized linear model (GLM). The regressors used for this analysis included a regressor of interest (RI) to model the hemodynamic response during the fMRI task as a double-gamma function. Multiple nuisance regressors were also included in the GLM, such as the temporal derivative of the RI, 24 regressors associated to realignment (Friston et al., 1996), and the detected outliers. The model output was a native functional space statistical map, which contained at each voxel the corresponding statistical z-value. Then, the LI was computed for each fMRI sequence as (Seghier, 2008):


$$\mathrm{LI}=\frac{\mathrm{Zva}l_{\mathrm{IH}}-\mathrm{Zva}l_{\mathrm{CH}}}{\mathrm{Zva}l_{\mathrm{IH}}+\mathrm{Zva}l_{\mathrm{CH}}}$$

Zval_IH_ and Zval_CH_ represent the sum of all positive z-values of the obtained statistical map within regions of interest (ROIs) of the ipsilesional and the contralesional hemisphere, respectively. In the present study, similar to related studies (Pantano et al., 1996; Szameitat et al., 2012; Rondina et al., 2016), the selected ROIs encompassed brain areas associated to motor activity, such as the pre-and post-central gyri, the supplementary motor area, the Rolandic operculum, the lentiform nucleus comprised by the putamen and the globus pallidus, the cerebellum, and the thalamus. These ROIs are defined in standard MNI152 space within the AAL3 anatomical atlas (Rolls et al., 2020), except for the thalamus, which is defined as a single ROI in the AAL2 atlas (Rolls et al., 2015).

A binary mask was created per hemisphere by combining, separately, the ipsilesional and contralesional individual ROIs of the previously defined anatomical regions, after considering each patient’s affected hemisphere. The contralesional cerebellum ROI was included in the ipsilesional binary mask, and vice versa, due to the decussation of the pyramidal tract at the cerebellum level (Vulliemoz et al., 2005). Lastly, the invRegF transformation was used to align the MNI152 binary masks to the statistical map’s native functional space through a non-linear registration. After summing the z-values within the masks, the LI could range from −1 to 1, indicating, respectively, a complete contralesional or ipsilesional hemispheric dominance when performing the motor task with either the unaffected or the affected hand. Additionally, an LI value of 0 represented a complete bilateral activation pattern.

#### 2.5.3 Ratio of fractional anisotropy

The rFA was used to assess the interhemispheric structural integrity of the white matter via DTI (Moura et al., 2019). Diffusion data analysis was performed in the FSL software. Preprocessing consisted of a correction of the eddy currents effect and of movement artifacts (Andersson and Sotiropoulos, 2016). Afterwards, the diffusion tensor was computed and used to obtain a voxelwise fractional anisotropy (FA) 3D map. FA tends to increase in areas with highly oriented fibers and adequately represents white matter structural integrity (Moura et al., 2019). Finally, the patient’s native FA map was registered to the standard MNI152 space through a non-linear transformation to the FMRIB58_FA_1mm template included in the FSL software (Jenkinson et al., 2012). The inverse transformation (invRegD) was then obtained by calculating the transformation’s inverse matrix. Then, the rFA was calculated as follows:


$$\mathrm{rFA}=\frac{FA_{\mathrm{IH}}}{FA_{\mathrm{CH}}}$$

Where FA_IH_ represents the mean FA value of the ipsilesional CST and FA_CH_ the analogue contralesional value. The hemispheric CST mean FA values were obtained by creating a CST mask for each hemisphere after combining the ROIs located at the superior corona radiate, the posterior limb of the internal capsule, the cerebral peduncle, and at the brainstem level of the CST, as defined in the JHU ICBM-DTI-81 White-Matter Labels atlas included in the FSL software (Mori et al., 2006). Then, the previously obtained invRegD transformation was used to transform the standard space CST masks to native diffusion space through a non-linear registration. Finally, the mean FA values of the ipsilesional and the contralesional CST were calculated as the average FA value within the corresponding mask. rFA values closer to 0 indicated lower white matter structural integrity of the ipsilesional CST compared to the contralesional tract, whereas values closer to 1 denoted a higher similarity in structural integrity between the ipsilesional and the contralesional CST.

All calculations in sections 2.5.1 to 2.5.3 were performed with MATLAB version 2021b (MathWorks Inc., Massachusetts, USA) and with the computational tools mentioned within these subsections.

#### 2.5.4 Motor-evoked potentials elicited by TMS

The CST integrity and excitability were measured using motor-evoked potentials elicited by single-pulse TMS. Studies were performed with a Magstim Rapid^2^ stimulator (Magstim Inc., Minnesota, USA) using a figure-of-eight coil. Motor evoked potentials (MEPs) were recorded with the device’s electromyography amplifier at a 1,500 Hz sampling frequency, with a bipolar configuration, from the first dorsal interosseous muscle of the affected and of the unaffected hand. Sessions of TMS started with an initial mapping of the sensorimotor cortex, to assess if patients had measurable MEPs. If patients’ MEPs could be elicited, then an adaptive method was used to compute the resting motor threshold (RMT) (Rossini et al., 2015; Julkunen, 2019). Subsequently, 30 MEPs were recorded from the hemisphere at the RMT. Afterwards, 30 MEPs were recorded at 120% of the RMT, followed by another 30 MEPs recorded at 140% of the RMT. This procedure was first performed for the contralesional and afterwards for the ipsilesional hemisphere. The mean peak-to-peak amplitudes of MEPs were computed separately for each hemisphere at 100, 120, and 140% of the RMT using an automatic recognition software (Tecuapetla-Trejo et al., 2021; Ortega-Robles et al., 2023). These amplitudes were analyzed to assess patients’ CST excitability and integrity in each hemisphere (Ortega-Robles et al., 2023).

### 2.6 Classification accuracy with the ReHand-BCI

Classification accuracy with the ReHand-BCI system was computed separately for the experimental and the control groups from the number of 1-s windows correctly and incorrectly classified as hand MI, or as rest with eyes open, and averaged across therapy sessions for each patient. Therefore, BCI performance was obtained for each group across all 30 therapy sessions and compared.

### 2.7 Statistical analysis

Differences between the experimental and control groups (intergroup differences) and between measurements in each group (intragroup differences) were assessed using nonparametric statistics after testing that data had non-gaussian distributions with Lilliefors tests, except for the classification accuracy with the BCI for which parametric statistics were used after confirming gaussian distributions. Intergroup differences of age and time since stroke lesion at T0 were evaluated using Mann–Whitney U tests. Intergroup differences between patients’ sex and lesioned hemisphere (left or right) at T0 were evaluated using Fisher’s exact test. Moreover, Mann–Whitney U tests were used to assess intergroup differences in primary and secondary outcomes separately for T0, T1, T2, and T3 measurements, and the effect size of these tests were estimated with the Pearson’s correlation coefficient (Maher et al., 2013; Tomczak and Tomczak-Łukaszewska, 2014). Besides, intragroup differences of primary and secondary outcomes, across T0, T1, T2, and T3 measurements, were assessed using Friedman tests with Bonferroni correction for post-hoc tests, and the effect size was estimated with Kendall’s W test value (Tomczak and Tomczak-Łukaszewska, 2014). For classification accuracy with the BCI, a two-sample t-test was used to assess differences between the experimental and the control groups, and Cohen’s d with Hedges’ correction was computed to assess effect size (Lakens, 2013).

Finally, MEPs could not be recorded in all patients’ measurements and hemispheres due to damage in the corticospinal tract or reaching the maximum stimulation intensity of the device. Therefore, only data of measurable MEPs were included in the TMS analysis of a given intervention measurement and hemisphere. This reduced the sample, and for this reason descriptive statistics were used to assess differences in MEP amplitudes recorded at 100, 120 and 140% of the RMT, across the intervention and in each hemisphere.

All inferential statistical tests were performed with a 95% confidence level using SPSS version 30 (IBM, New York, USA).

## 3 Results

### 3.1 Patients

**Figure 2:**
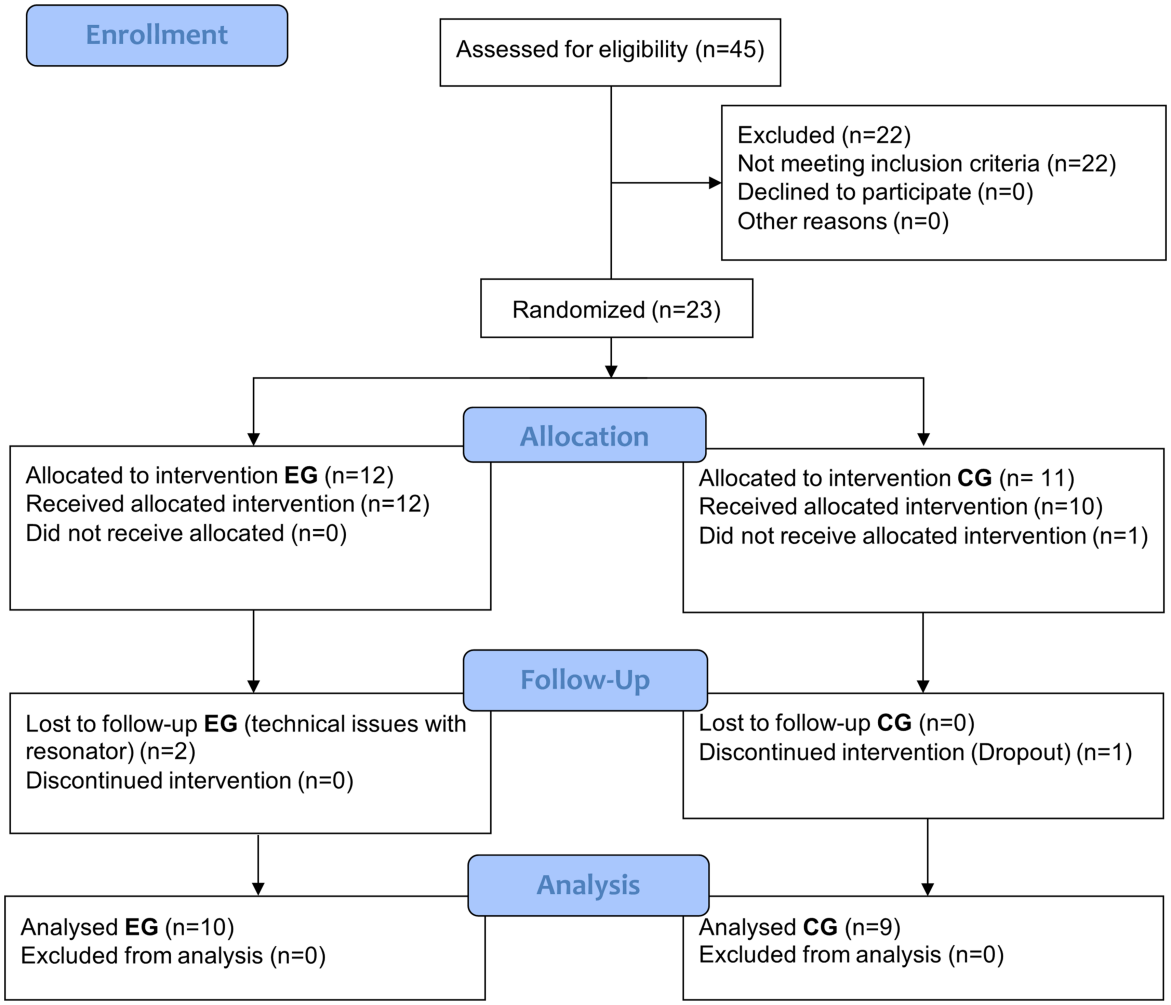
CONSORT flow diagram for the experimental group (EG) and the control group (CG).

Recruitment spanned from March 16th, 2021, to January 16th, 2024, with the last follow-up measurements performed on June 24th, 2024. Forty-five patients were assessed for eligibility, with 22 patients not meeting inclusion criteria. Twenty-three patients were included in the study. Twelve patients were allocated in the experimental group and 11 in the control group after randomization. During the study, no adverse effects were associated with the intervention. One patient in the control group decided to withdraw from the study citing time constraints. For 2 patients in the experimental group, complete outcome assessments were not performed due to technical issues that impeded the acquisition of magnetic resonance studies for these patients, thus, they were not included in the study’s final analysis. Therefore, 10 patients were included for the final analysis in the experimental group and 9 in the control group. Participant recruitment ended in January 2024 due to the conclusion of study funding; therefore, the target sample size of 30 patients was not reached. Figure 2 depicts patients’ enrollment.

Table 1 shows patients’ clinical and demographic characteristics. Fourteen males (6 in the experimental group and 8 in the control group) and 5 females (4 in the experimental and 1 in the control group) concluded the study. Baseline comparisons revealed no statistically significant differences between the experimental and control groups regarding age, time since stroke onset, sex, or lesioned hemisphere. At the start of the study six patients were in the late subacute stage of stroke, 3 to 6 months, while the others were in the chronic stage, more than 6 months (Bernhardt et al., 2017). Ischemic stroke comprised most of the sample, with only one patient with a mixed stroke included in the study. Detailed patients’ demographic and clinical features, as well as the measurements of primary and secondary outcomes, can be found in Supplementary Table S2.

**Table 1:** Patients’ demographic and clinical characteristics.

ID (Assigned group)	Age (years)	Sex	Time since stroke onset (months)	Stroke type, lesion type	Ipsilesional hemisphere	Lesion location, ASPECTS
P1 (EG)	48	Female	13	Ischemic, Cortical	Right	Temporal–parietal, 30%
P2 (EG)	47	Male	4.6	Hemorrhagic, Cortical–subcortical	Right	Temporal, NA
P3 (CG)	66	Male	17.7	Hemorrhagic, Subcortical,	Left	Temporal–parietal–frontal, NA
P4 (EG)	41	Female	8.8	Ischemic, Cortical–subcortical	Right	Temporal, 40%
P5 (CG)	69	Male	7.5	Ischemic-Hemorrhagic, Cortical–subcortical	Right	Frontal-temporal, 70%
P6 (CG)	59	Male	3.8	Ischemic, Cortical–subcortical	Right	Frontal-temporal–parietal, 90%
P7 (EG)	56	Male	4.9	Hemorrhagic, Subcortical	Right	Basal ganglia, NA
P8 (EG)	27	Female	14.2	Hemorrhagic, Subcortical	Left	Basal ganglia-thalamus, NA
P9 (CG)	64	Male	19.3	Ischemic, Cortical–Subcortical	Left	Parietal, 10%
P10 (EG)	66	Male	14.4	Ischemic, Subcortical	Right	Periventricular, NA
P11 (EG)	49	Male	20.5	Hemorrhagic, Subcortical	Right	Putamen, NA
P12 (EG)	18	Female	22.2	Ischemic, Cortical–subcortical	Right	Temporal, 30%
P13 (CG)	60	Female	16.8	Ischemic, Cortical–subcortical	Right	Temporal, 40%
P14 (EG)	63	Male	10.9	Ischemic, Cortical–subcortical	Left	Temporal–parietal, 40%
P15 (CG)	58	Male	12.5	Ischemic, Subcortical	Right	Pons, medulla oblongata, NA
P16 (CG)	21	Male	5.7	Ischemic, Subcortical	Right	Anterior choroidal artery
P17 (CG)	43	Male	5.3	Hemorrhagic, Subcortical	Right	Periventricular
P18 (EG)	63	Male	13.2	Ischemic, Subcortical	Left	Periventricular, 10%
P19 (CG)	62	Male	4.4	Ischemic, Cortical–subcortical	Right	Frontal–parietal, temporal, 70%

Age is specified in years, and the time since stroke onset is in months. ASPECTS scale was used to estimate the percentage of infarct in regions related to the middle cerebral artery. If the lesion type could not be assessed with ASPECTS, then it was marked as NA. EG, Experimental Group. CG, Control Group.

### 3.2 Primary outcome measures

**Figure 3:**
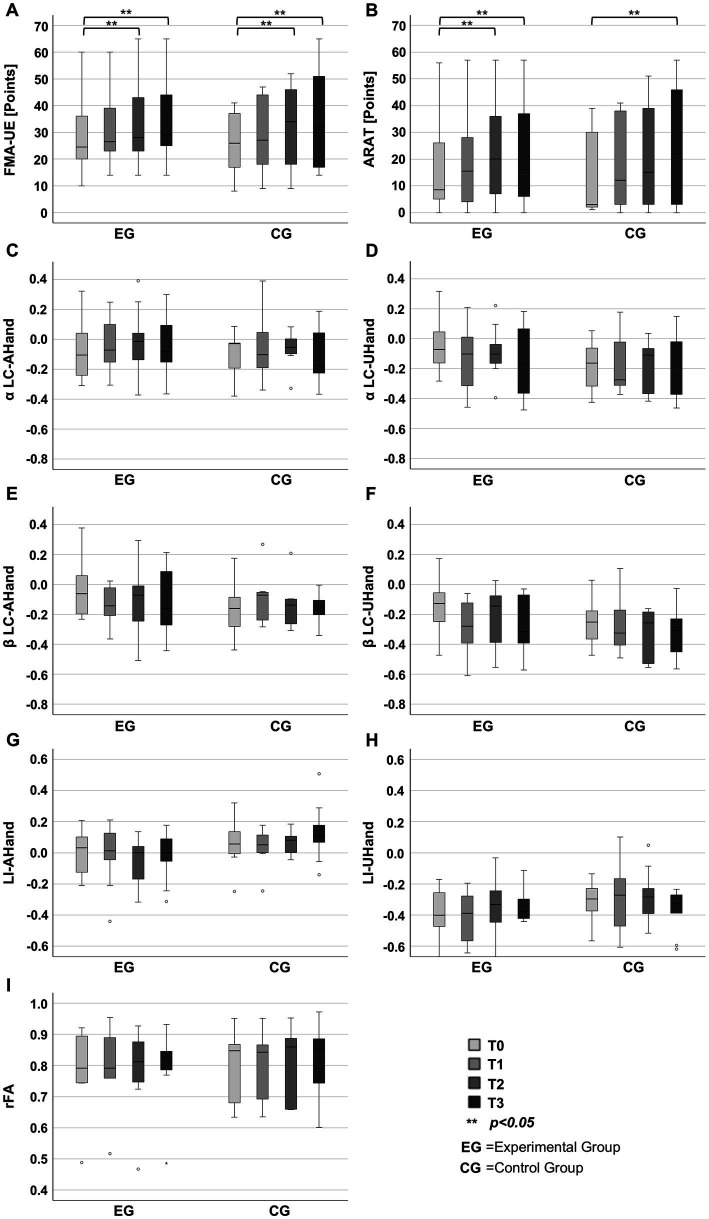
Outcomes measured at baseline (T0), intermediate (T1), post-treatment (T2), and follow-up (T3). **(A)** Fugl-Meyer Assessment for the Upper Extremity (FMA-UE). **(B)** Action Research Arm Test (ARAT). **(C)** Laterality coefficient (LC) computed from alpha during the affected hand movement intention (αLC-AHand). **(D)** LC computed from alpha during unaffected hand movement (αLC-UHand). **(E)** LC computed from beta during the affected hand movement intention (βLC-AHand). **(F)** LC computed from beta during unaffected hand movement (βLC-UHand). **(G)** Laterality index (LI) during the affected hand movement intention (LI-AHand). **(H)** LI during the unaffected hand movement (LI-UHand). **(I)** Ratio of fractional anisotropy (rFA).

Figures 3A,B show clinical measures for each group across the intervention. Table 2 shows statistical significance and effect sizes for intergroup and intragroup comparisons. There were no statistically significant differences between the experimental and control groups’ FMA-UE scores in any of the intervention measurements, in addition, small effect sizes (Maher et al., 2013) were observed. Significant intragroup differences and a large effect size (Maher et al., 2013), were observed across the intervention measurements of FMA-UE for the experimental group according to a Friedman test. Post-hoc comparisons showed significant differences of FMA-UE scores in the experimental group between T0 (24.5[20,36]) and T2 (28[23, 43]) scores and between T0 (24.5[20,36]) and T3 (29[25,44]). Intragroup differences were also observed across the intervention FMA-UE scores in the control group, according to a Friedman test with a large effect size. Post-hoc comparisons showed significant differences of FMA-UE scores between T0 (26[16,37.5]) and T2 (34[17.3,46.5]) scores, and between T0 (26[16,37.5]) and T3 (32[16.8,52]) scores.

**Table 2:** Intergroup and intragroup statistical comparisons.

Outcome variables	Intergroup comparisons				Intragroup comparisons	
Measurement/Group	T0	T1	T2	T3	Experimental group	Control group
FMA-UE	*p* = 0.656 *r =* −0.028	*p* = 1 *r = −*0.038	*p* = 0.656 *r =* 0.019	*p* = 0.656 *r =* 0.019	*p* < 0.001 *W* = 0.629 Post-hoc tests: T0 with T1 *p* = 0.5 T0 with T2 *p* = 0.011 T0 with T3 *p* = 0.001 T1 with T2 *p* = 0.995 T1 with T3 *p* = 0.226 T2 with T3 *p* = 1	*p* = 0.001 *W* = 0.597 Post-hoc tests: T0 with T1 *p* = 0.497 T0 with T2 *p* = 0.008 T0 with T3 *p* = 0.003 T1 with T2 *p* = 0.865 T1 with T3 *p* = 0.497 T2 with T3 *p* = 1
ARAT	*p* = 1 *r* = −0.056	*p* = 1 *r* = −0.028	*p* = 1 *r* = −0.094	*p* = 0.656 *r* = 0.009	*p* = 0.001 *W* = 0.547 Post-hoc tests: T0 with T1 *p* = 0.278 T0 with T2 *p* = 0.034 T0 with T3 *p* = 0.002 T1 with T2 *p* = 1 T1 with T3 *p* = 0.714 T2 with T3 *p* = 1	*p* = 0.011 *W* = 0.416 Post-hoc tests: T0 with T1 *p* = 1 T0 with T2 *p* = 0.865 T0 with T3 *p* = 0.037 T1 with T2 *p* = 1 T1 with T3 *p* = 0.407 T2 with T3 *p* = 1
αLC-AHand	*p* = 0.656 *r* = 0.037	*p* = 1 *r* = 0.076	*p* = 0.327 *r* = 0.076	*p* = 0.656 *r* = 0.15	*p* = 0.516 *W* = 0.076	*p* = 0.316 *W* = 0.131
αLC-UHand	*p* = 0.37 *r* = 0.319	*p* = 0.37 *r* = 0.093	*p* = 1 *r* = 0.15	*p* = 1 *r* = 0.112	*p* = 0.373 *W* = 0.104	*p* = 0.706 *W* = 0.052
βLC-AHand	*p* = 0.37, *r* = 0.356	*p* = 0.656 *r* = 0.056	*p* = 0.37 *r* = 0.225	*p* = 1 *r* = 0.056	*p* = 0.696 *W* = 0.048	*p* = 0.269 *W* = 0.146
βLC-UHand	*p* = 0.07 *r* = 0.337	*p* = 1 *r* = 0.056	*p* = 0.37 *r* = 0.337	*p* = 1 *r* = 0.075	*p* = 0.118 *W* = 0.196	*p* = 0.129 *W* = 0.21
LI-AHand	*p* = 0.656 *r* = 0.131	*p* = 0.179 *r* = 0.187	*p* = 0.656 *r* = 0.3	*p* = 0.179 *r* = 0.393	*p* = 0.516 *W* = 0.076	*p* = 0.769 *W* = 0.042
LI-UHand	*p* = 0.179 *r* = 0.262	*p* = 0.179 *r* = 0.3	*p* = 0.656 *r* = 0.15	*p* = 0.656 *r* = 0.094	*p* = 0.392 *W* = 0.1	*p* = 0.506 *W* = 0.86
rFA	*p* = 0.179 *r* = 0.075	*p* = 0.179 *r* = 0.056	*p* = 0.656 *r* = 0.112	*p* = 0.179 *r* = 0.169	*p* = 0.045 *W* = 0.268 Post-hoc tests: T0 with T1 *p* = 1 T0 with T2 *p* = 0.714 T0 with T3 *p* = 0.092 T1 with T2 *p* = 0.995 T1 with T3 *p* = 0.146 T2 with T3 *p* = 1	*p* = 0.068 *W* = 0.264

The significance value (p) and the effect size (*r* = Pearson’s correlation coefficient for intergroup comparisons with Mann–Whitney U tests, or W = Kendall’s W test value for intragroup comparisons with the Friedman test) were computed for each test. Post-hoc tests’ significance are shown if Friedman tests implied significant intragroup differences.

Table 2, Figures 3C,D show ARAT comparisons for intergroup and intragroup tests. There were no statistically significant differences between the experimental and control groups’ ARAT scores in any of the intervention measurements, in addition, small effect sizes were observed for these comparisons. Intragroup differences and large effect size (Maher et al., 2013) were observed across the intervention measurements of ARAT for the experimental group, according to a Friedman test. Post-hoc comparisons showed significant differences of ARAT scores in the experimental group between T0 (8.5[5, 26]) and T2 (20[7, 36]) scores, and between T0 (8.5[5, 26]) and T3 (15[2.5, 40.8]) scores. Intragroup differences and a medium effect size were observed across the ARAT scores in the control group, according to a Friedman test. Post-hoc comparisons only showed significant differences of ARAT scores between T0 (3[1.8, 30.5]) and T3 (22[2.5, 47.3]) scores.

### 3.3 Secondary outcome measures

Table 2 and Figures 3E–I show secondary outcomes measures and comparison statistics for each group across the intervention. There were not statistically significant intergroup differences at any measurement, T0, T1, T2, or T3, in any of the assessed secondary outcomes. Intergroup comparisons had small to medium effect sizes. There were also not statistically significant intragroup differences in the LC or LI during hand movement-related tasks, in both the experimental and the control groups, in addition to presenting small to medium effect sizes. In the experimental group, a statistically significant difference and a moderate effect size was observed in the rFA, however, after Bonferroni correction, post-hoc tests did not show significant differences between measurements. In the control group, the rFA did not had statistically significant differences across measurements, and had a medium effect size.

**Figure 4:**
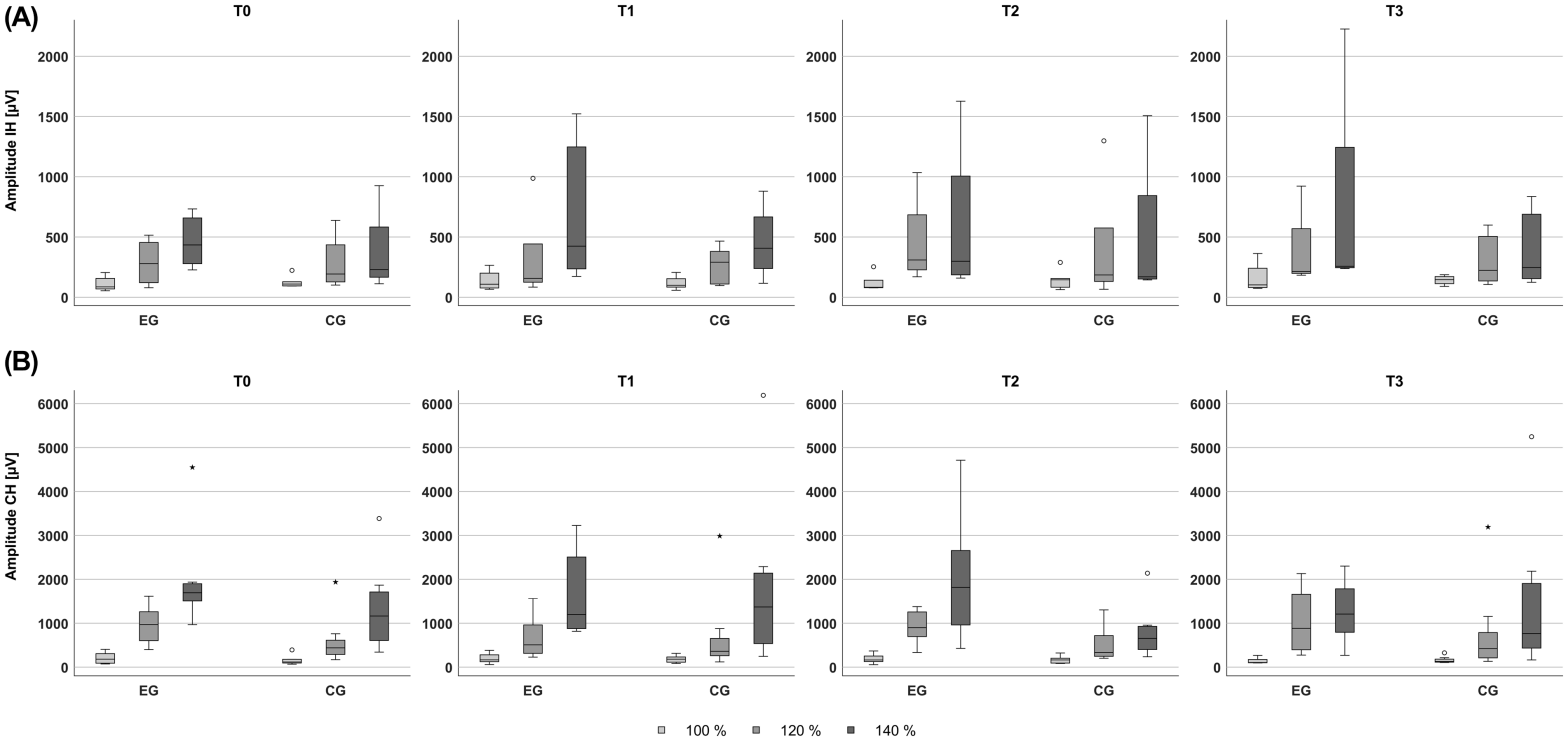
Amplitude of motor evoked potentials elicited by transcranial magnetic stimulation using 100, 120 and 140% of the resting motor threshold, across the baseline (T0), intermediate (T1), post-treatment (T2), and follow-up (T3) sessions, for the experimental (EG) and control (CG) groups. **(A)** Amplitudes recorded from ipsilesional hemisphere (IH) stimulation. **(B)** Amplitudes recorded from contralesional hemisphere (CH) stimulation.

Figure 4 shows MEP amplitudes of each group across the intervention and at the 3 elicited stimulation intensities using TMS, per hemisphere. Five patients in the experimental group and six in the control group presented measurable MEPs in the ipsilesional hemisphere at T0. At T2 the same five patients in the experimental and six patients in the control group had measurable MEPs in the ipsilesional hemisphere. At T3 four patients in the experimental group and four patients in the control group had measurable MEPs in their ipsilesional hemisphere. All median values of recorded MEPs were higher than 50 uV. An increase of MEP amplitude was observed with an increase of stimulation output in the experimental group and control groups across all measurements and in both hemispheres. In the ipsilesional hemisphere, the distribution of MEP amplitudes at 120 and 140% of the RMT, encompassed higher values in the experimental group, compared to the control group at T2 and T3. In the contralesional hemisphere, the distribution of MEP amplitudes at 140% of the RMT had higher values at T2 in the experimental group, while they had their lowest values at T2 in the control group.

**Figure 5:**
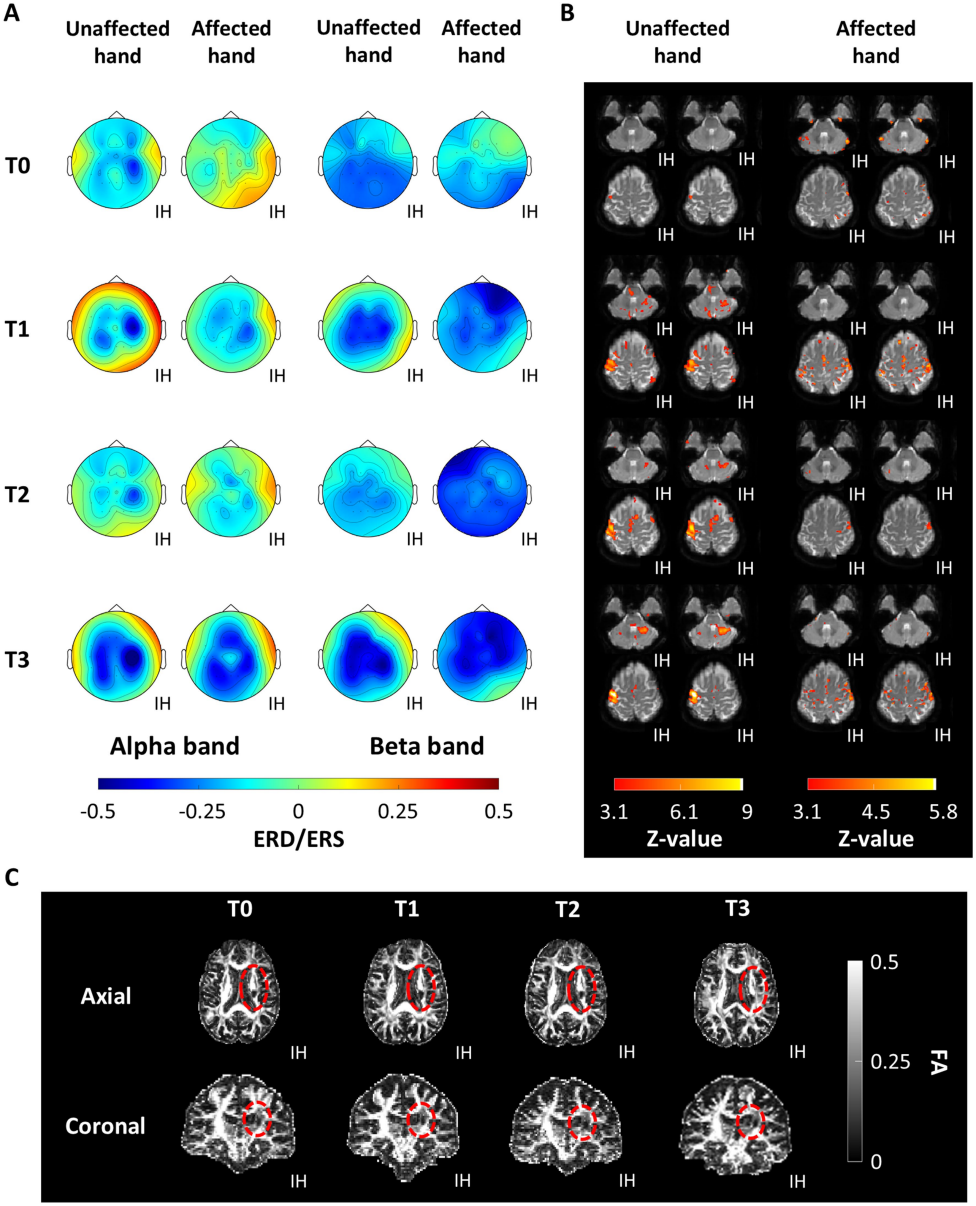
Functional and structural features of patient P11 within the experimental group across the baseline (T0), intermediate (T1), post-treatment (T2), and follow-up (T3) measurements. **(A)** Cortical activity assessed by event-related desynchronization/synchronization (ERD/ERS). **(B)** Brain activity measured with functional magnetic resonance. **(C)** Fractional anisotropy (FA) computed from diffusion tensor imaging, the region encompassing the lesion is encircled in red. The ipsilesional hemisphere (IH) is indicated for all plots.

Figure 5 displays the statistically significant ERD/ERS, fMRI activation, and FA maps of a representative patient within the experimental group across the intervention to show the features from which the LC, LI, and rFA were computed. Figure 5A shows how contralesional alpha ERD was observed across the intervention during the unaffected hand movement, while bilateral ERD was consistently observed in beta. In addition, alpha and beta ERD were observed in the ipsilesional motor cortex across all the intervention measurements during MI of the affected hand. Figure 5B depicts fMRI activation maps during the unaffected hand movement and the affected hand MI. The unaffected hand motor tasks produced activation patterns around motor control areas. Affected hand motor tasks produced more variable activation patterns but were concentrated in the ipsilesional hemisphere at T2. Figure 5C shows FA maps of the patient depicting the stroke lesion in the posterior limb of the right hemisphere’s internal capsule. However, it is difficult to determine structural changes qualitatively across assessment sessions.

**Figure 6:**
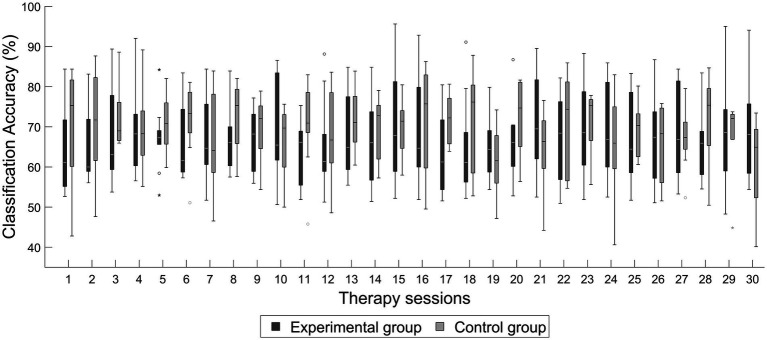
Average classification accuracy across therapy sessions of the experimental and control groups.

Figure 6 shows the classification accuracies computed for each of the 30 therapy sessions for the experimental and the control groups. There were not statistically significant differences between the average classification accuracy obtained by the experimental group 67.3% ± 8.97% and the perceived by the control group 69.28% ± 4.1% (*p* = 0.557, *d* = −0.263).

## 4 Discussion

Sensorimotor upper extremity function measured with the FMA-UE did not show significant differences between the experimental and the control group. However, patients in both groups had higher FMA-UE scores after each intervention, with significant increases in FMA-UE scores at the post-treatment and follow-up compared to baseline measurements. In addition, the average change of FMA-UE scores observed at post-treatment in the experimental group was 4.5 ± 2.6 points, while the average change of FMA-UE in the control group was 6.2 ± 4.1. This recovery was noticeable if it is taken into account that averaged FMA-UE scores at baseline in both groups were in the range of a poor sensorimotor capacity of the upper extremity (27.6 ± 13.6), implying a severe disability, with recovery being less likely in these patients (Hoonhorst et al., 2015). Besides, in the experimental and control groups sensorimotor recovery was within the range of 5.25 FMA-UE points, which is reported as the minimal clinically important difference (MCID) (Page et al., 2012).

Furthermore, the observed change of FMA-UE scores in both groups were within the range of sensorimotor upper extremity recovery reported in several BCI studies for upper extremity stroke rehabilitation: (4.55 ± 6.1) (Ang et al., 2015a), (7.2 ± 2.3) (Ang et al., 2014), (3.4 ± 2.2) (Ramos-Murguialday et al., 2013), (4.7 ± 12) (Frolov et al., 2017), (4.68 ± 4.92) (Sebastián-Romagosa et al., 2020a), (7.87 ± 2.4) (Kim et al., 2016) and (3.8 ± 5.3) (Cheng et al., 2020). Therefore, it can be implied that the BCI intervention with the ReHand-BCI system, comprising robotic feedback, can significantly improve upper extremity sensorimotor function in stroke patients. It can also be suggested that a sham intervention seems to have similar effects in sensorimotor function compared to actual BCI robotic feedback. Qu et al. also assessed that sensorimotor function recovery measured with FMA-UE, was not significantly different in studies that compared BCI therapies that had robotic feedback, with therapies that only applied robotic feedback (Qu et al., 2024). They hypothesized that the lack of observed clinical differences was due to both interventions promoting upper sensorimotor function combined with the limited sample of the studies. Therefore, the absence of significant differences between BCI and sham-BCI therapies in the current study could have been caused by the same reasons remarked by Qu et al. in their analysis.

Functional performance of the upper extremity, assessed with the ARAT, was not significantly different between the experimental and the control group. However, patients in the experimental group had a higher ARAT score at the end of the intervention, with a significant increase and a large effect size at the post-treatment and follow-up compared to baseline measurements. On the other hand, patients in the control group only had a significant increase of ARAT scores at follow-up, compared to baseline. This implied that the effects on functional performance in stroke can be higher if stroke patients control the hand movement provided with a robotic device using their paralyzed hand MI. Changes of ARAT at post-treatment observed in the experimental (8.4 ± 7.6) and control (7 ± 8.9) groups were within the range of the MCID (5.7) (Van Der Lee et al., 2001). As with FMA-UE, this recovery was notable due to patients in both groups presenting a poor functional upper extremity capacity (Hoonhorst et al., 2015) due to having an average ARAT score of 14.9 ± 16.3 at baseline. Therefore, it can be implied that therapies comprising actual BCI control and sham BCI with the ReHand-BCI system can have noticeable clinical effects regarding upper extremity functional performance, with a tendency towards higher recovery achieved with BCI intervention. ARAT is not as widely reported in BCI studies applied to stroke rehabilitation, compared to FMA-UE, but the observed results were within the range of the 2 points of ARAT gains reported in the studies of Frolov et al. (2017) and Remsik et al. (2018). It is possible that the more pronounced changes observed in the experimental group in ARAT compared to FMA-UE, were due to ARAT assessing functional components of the upper extremity, including the ability of patients to perform grasping tasks, which was the type of task that therapy with the ReHand-BCI focused on.

Follow-up measurements of more than 12 weeks after a BCI intervention for upper extremity rehabilitation are scarcely reported within the literature (Ang et al., 2014; Biasiucci et al., 2018; Ramos-Murguialday et al., 2019). In this study, patients were assessed at 18 weeks after ending the BCI intervention, with upper extremity conventional therapy not restricted in this period. However, patients’ recovery was lower, or their function even decreased when compared to the recovery observed during the 6 weeks that lasted the intervention period. This suggests that the gains of sensorimotor function and functional performance observed during the intervention were likely elicited by therapy-induced neuroplasticity effects and not spontaneous recovery, providing much needed evidence of the short and long-term effects of a BCI or sham-BCI intervention.

Hemispheric dominance during unaffected hand movement or affected hand MI, measured with the LC, did not show significant intergroup or intragroup differences. Only the LC in alpha during affected hand MI in the experimental group tended to higher values, closer to 0, between baseline and post-treatment measurements, with the median LC dropping to almost baseline values at follow up. This implied that at the onset of the BCI intervention patients had a more pronounced contralesional dominance during affected hand MI, but by the end of the intervention patients had a more symmetrical activation and returned to a more pronounced contralesional activation months after the intervention. Sebastián-Romagosa et al. (2020b) reported an association between an alpha LC closer to 0, computed during the affected hand motor imagery, with higher sensorimotor function of the upper extremity in stroke patients that underwent 25 sessions of BCI therapy. Therefore, the observed tendency of LC in alpha during patients’ affected hand MI in the experimental group of the present study, could have reflected that a more balanced hemispheric dominance was also associated with a recovery of upper extremity sensorimotor function after the BCI intervention. Interestingly, the observed decrease of median alpha LC during affected hand MI in the follow-up measurement could have reflected that the neuroplasticity effects of the experimental intervention were not present after it was concluded, as suggested by most of FMA-UE and ARAT follow-up measurements. An analysis of session-to-session LC changes could bring more insight into specific neuroplasticity effects of the interventions and is proposed as future work.

Regarding hemispheric dominance during affected and unaffected hand motor tasks computed with the LI derived from fMRI. Patients generally exhibited mild ipsilesional activations during affected hand MI, and moderate contralesional activations were present during the unaffected hand movement. This was expected due to the affected CST making ipsilesional activations less likely during movement-related tasks with the affected hand in stroke patients (Cunningham et al., 2015). During the recovery of upper extremity function in stroke patients, it has been reported that the LI shows changes towards a higher ipsilesional dominance elicited with affected hand movements, especially in the premotor and sensorimotor cortex (Tang et al., 2015). However, in the present study, no significant intergroup or intragroup differences regarding hemisphere dominance were measured with the LI in any of the assessed measurements. The study of Young et al. (2015) also assessed the LI during a BCI intervention in stroke and observed that the LI changed towards the contralesional hemisphere across sessions, while Ramos-Murguialday et al. (2013) and Yuan et al. (2020) reported significant changes of LI toward the ipsilesional hemisphere after their BCI intervention. A possible reason for the observed absence of significant LI differences could be the limited sample of 10 patients per group, lower than the 16 patients in Young et al. (2015) study, 16 per group in the study of Ramos-Murguialday et al. (2013), and the 12 patients per group in Yuan et al. (2020) study. Thus, the sample size could not have allowed to compensate for the variability that has been reported with this technique in ischemic stroke patients, which has been hypothesized to be caused by dynamic bilateral hemispheric functional reorganization of networks (Yu et al., 2024).

The interhemispheric structural integrity of the white matter was assessed using rFA derived from DTI. Although the values of rFA were not significantly different between groups, the rFA in the experimental group significatively tended to have higher median values across the intervention and follow-up measurements, however, the effect size was moderate. A higher rFA reflects a higher similarity of structural integrity between the ipsilesional and the contralesional CST and has been associated with higher upper extremity motor function (Yuan et al., 2020; Xia et al., 2021; Shaheen et al., 2022). Yuan et al. also assessed rFA during a BCI intervention and observed a higher ipsilesional CST integrity that was related to motor function improvement (Yuan et al., 2020). On the other hand, Young et al. observed a relationship between a higher contralesional CST integrity, measured via FA, with higher functional performance assessed with the ARAT in 10 stroke patients during a BCI intervention, and hypothesized that this was due to most of their patient sample having a severely affected CST (Young et al., 2016). Therefore, the observed increase in ipsilesional white matter integrity of the CST, across the BCI and sham-BCI interventions, are likely the consequence of elicited neuroplasticity processes. These neuroplasticity processes seem to focus on remodeling white matter, mainly in the ipsilesional hemisphere, which highlights the importance of ipsilesional white matter integrity for stroke neurorehabilitation, as has been previously stated in the literature (Pinter et al., 2020). Few studies have assessed rFA from DTI during a BCI intervention of the upper extremity (Young et al., 2016; Caria et al., 2020; Yuan et al., 2020), making the observed findings in this study a valuable contribution that supports the hypothesis that BCI, and even sham-BCI, elicited mechanisms of motor recovery can be mainly explained by a higher ipsilesional CST white matter integrity.

CST integrity and excitability were assessed with TMS. However, nearly half of the patients in each group had a severely affected CST, as indicated by the absence of measurable MEPs in the ipsilesional hemisphere. This highlights the importance of the observed recovery of upper extremity motor function during the BCI and sham-BCI interventions, since a severely affected CST in stroke is associated with a poor motor function prognosis (Stinear et al., 2017; Shaheen et al., 2022). In the ipsilesional hemisphere, a higher corticospinal excitability was observed in both groups at post-treatment, which remained at follow-up only in the experimental group, implied by the higher values of MEP amplitudes at 120 and 140% of the RMT. This suggested that immediately after receiving 30 intervention sessions patients that had a BCI or sham-BCI therapy presented more noticeable CST excitability in the ipsilesional hemisphere, but it only prevailed after 18 weeks of receiving the intervention in the group that had BCI therapy. Furthermore, at follow-up, only one patient’s ipsilesional MEPs could not be measured compared to two patients in the control group, further implying more lasting effects in corticospinal tract integrity with a BCI therapy. This is in line with the trend of increased ipsilesional corticospinal integrity measured using the rFA in the experimental group, supporting the hypothesis that the observed functional recovery could be attributed, at least in part, to enhanced ipsilesional corticospinal integrity and excitability in the experimental group.

Conversely, in the contralesional hemisphere, patients in the experimental group presented higher values of MEP amplitude at 120 and 140% of the RMT immediately after the intervention and at follow-up, compared to the control group. Therefore, it can be suggested that the BCI intervention elicited higher contralesional corticospinal excitability compared to sham-BCI. A higher contralesional corticospinal excitability has been associated with compensatory neuroplasticity mechanisms in stroke, mainly observed in patients with severely affected ipsilesional CST (McPherson et al., 2018; Williamson et al., 2024). Since few clinical trials comprising BCI interventions for upper extremities in stroke have applied TMS for CST assessment (Pichiorri et al., 2015; Brunner et al., 2024), the observed findings are of importance since they imply that both ipsilesional and contralesional corticospinal excitability is enhanced during a BCI intervention.

The BCI performance shown by the experimental group and the one perceived by the control group were similar, implying that patients received comparable feedback in the BCI therapy and in the sham-BCI intervention. This is important, since these performances with the BCI implied that patients in both groups had a comparable engagement with the BCI system due to receiving a similar amount of feedback. Furthermore, the BCI performance of 67.3 ± 8.97% classification accuracy achieved by the experimental group also suggested that most patients were able to control the system within the range of the BCI control achieved by stroke patients in other studies. For example, in the study of Ang et al. (2011) the average BCI performance achieved by stroke patients was 74%, while in the study of Fu et al. (2023) the average classification accuracy was of 70.7%. Therefore, the degree of BCI control in the experimental group was the one that could have been expected in a stroke population.

Due to primary motor outcomes of sensorimotor and functional performance not showing significant intergroup differences, it can be stated that the hypothesis of a higher recovery elicited with the BCI system compared to a sham-BCI intervention, was not accepted in the present study. Ipsilesional cortical activity, ipsilesional corticospinal tract integrity, and corticospinal tract excitability were not significantly higher in the experimental group. However, ipsilesional cortical activity measured with alpha LC and ipsilesional corticospinal tract integrity measured with the rFA and TMS tended to be more pronounced across the intervention in the experimental group. Therefore, it is implied that neuroplasticity mechanisms involving the ipsilesional hemisphere are likely related to the increased sensorimotor and functional performance of the upper extremity observed with the BCI intervention. The lack of significant differences, between a therapy based on a BCI-triggered robotic feedback compared to a sham-BCI random robotic feedback, was also acknowledged in the analysis reported by Qu et al. (2024). Since the BCI performance achieved by the experimental group was not significantly different compared to the BCI performance perceived by the control group, then the absence of intergroup differences was likely caused by the most important of the study’s limitations, the limited sample size. Prior evidence indicates that BCI interventions for post-stroke upper limb rehabilitation generally produce medium effect sizes (Bai et al., 2020). Therefore, it is plausible that the current study was underpowered to detect such intergroup effects. A larger sample size would be necessary to more rigorously evaluate differential efficacy and underlying neuroplastic changes between BCI and sham-BCI therapies. Another limitation of the study was the heterogeneity of stroke lesions among participants, which included cortical, subcortical, or cortical–subcortical lesions. In addition, while most participants were in the chronic stage of stroke, a few were in the late subacute stage. This variability in lesion location and stroke stage may have contributed to differences in recovery profiles, as patients with different stroke etiologies and stages often follow distinct recovery trajectories (Bernhardt et al., 2017). However, the motor sequelae observed in patients at the onset of the interventions was homogeneous, which could have attenuated the effect of variability of stroke lesions in motor recovery. Furthermore, late subacute and chronic stroke patients are the most likely candidates to receive BCI therapies in healthcare facilities, and previous studies have included stroke patients with mixed chronicity (Mane et al., 2020).

The present study provided some relevant insights for assessing the clinical and neuroplasticity effects of BCI-based upper extremity therapies in stroke. To the authors’ knowledge, this is one of the controlled BCI intervention studies that has provided the highest number of therapy sessions (30 sessions), with previous studies ranging from 12 to 20 sessions of BCI therapy (Várkuti et al., 2012; Ramos-Murguialday et al., 2013, 2019; Ang et al., 2014, 2015a; Frolov et al., 2017; Cheng et al., 2020; Wang et al., 2024). Moreover, this is the only study that has assessed sensorimotor and functional clinical performance, in addition to hemispheric dominance from EEG, as well as hemispheric dominance via fMRI, white matter integrity within the CST using DTI, and CST integrity and excitability with TMS. Thus, the study provides new evidence of the neuroplasticity mechanisms elicited by a BCI intervention, from a range of physiological measurements that have not been assessed in conjunction before. Another contribution of this study is the follow-up assessment of clinical and physiological features of patients several months after the intervention, which are rarely reported in BCI-related clinical trials, despite being highly relevant for understanding BCI-associated neuroplasticity effects (Bai et al., 2020). Therefore, the present study provides relevant evidence regarding the clinical efficacy and neuroplasticity effects of therapy with the ReHand-BCI system, implying that even a sham-BCI intervention can lead to significant function improvements in stroke. Finally, it can be inferred that the sensorimotor clinical effects of a long intervention with the ReHand-BCI are similar to those of its sham counterpart. However, this was not the case with functional performance, as well as for several physiological effects, therefore, there seems to be advantages of providing close-loop feedback with the system. Future studies with the ReHand-BCI system could involve stratifying patients depending on features such as the type of lesion or degree of upper extremity impairment to provide a higher therapy intensity depending on these features, or analyzing which group of patients could benefit more with a BCI intervention.

Clinical and a wide variety of physiological effects elicited by a BCI intervention using the ReHand-BCI system were compared to a sham-BCI intervention across 30 rehabilitation sessions in this clinical trial. Even though significant differences in upper extremity motor function recovery were not observed between the BCI and sham-BCI therapies, patients in both groups presented a clinically meaningful recovery during the interventions. This recovery was likely attributed to post-treatment enhancement of ipsilesional corticospinal structural integrity, a more balanced interhemispheric white matter integrity, and pronounced bilateral corticospinal excitability. Although the number of recruited patients was limited, to the authors’ knowledge, this is the first controlled clinical trial that assessed such a wide range of physiological effects across a long BCI intervention. The observed clinical and physiological effects of the BCI intervention allow to infer that closed-loop feedback can promote functional and structural changes that are not equal to a sham-BCI but can present a higher or similar effect in upper extremity recovery.

## Data Availability

The original contributions presented in the study are included in the article/Supplementary material, further inquiries can be directed to the corresponding author.

## Supplementary material

Supplementary Video S1 Shows the elements of the ReHand-BCI system. The video also shows a stroke patient controlling the ReHand-BCI using motor intention of the paralyzed upper extremity during a therapy session with the system.

Supplementary Table S1 Excel file that contains the dataset with clinical and neuroplasticity-related variables per patient in each of the four intervention measurements. The sheet named “Data” shows patients variables, while the sheet named “Coding” describes the codification used for each of the clinical and neuroplasticity-related variables.
